# An early implementation assessment of Ontario’s Healthy Kids Community Challenge: results from a survey of key stakeholders

**DOI:** 10.1186/s12889-019-7704-2

**Published:** 2019-11-27

**Authors:** Michelle M. Vine, Jocelyn W. Jarvis, Eunice Chong, Rachel E. Laxer, Adam Ladak, Heather Manson

**Affiliations:** 1 Department of Health Promotion, Chronic Disease and Injury Prevention, Public Health Ontario, Suite 300 – 480 University Avenue, Toronto, ON M5G 1V2 Canada; 2 School of Public Health and Health Systems, University of Waterloo, 200 University Avenue West, Waterloo, ON N2L 3G1 Canada; 3 Dalla Lana School of Public Health, University of Toronto, Health Sciences Building, 155 College Street, 6th Floor, Toronto, ON M5T 3M7 Canada

**Keywords:** Health promotion intervention, EPODE model, Obesity prevention, Process evaluation, Community-based program

## Abstract

**Background:**

In Ontario Canada, the Healthy Kids Community Challenge (HKCC) is a program intended to reduce the prevalence and prevent childhood overweight and obesity through community-based initiatives to improve health behaviours. Guided by the RE-AIM framework and Durlak and DuPre’s Ecological Framework for Understanding Effective Implementation, the evaluation focused on two objectives: 1) to describe the organization of the program at the community level; and, 2) to identify opportunities for improvement through an early assessment of factors contributing to implementation.

**Methods:**

Participants (*n* = 320) – members of the HKCC local steering committee, including the local project manager – completed a cross-sectional survey using SurveyMonkey and descriptive statistics were calculated. A sample (20%) of qualitative open-ended responses was thematically analyzed.

**Results:**

Results indicated strong respondent agreement that the HKCC enhanced individual knowledge of access to health-promoting programs (88.3%) and messaging regarding healthy behaviours for healthy kids, with less for its effectiveness in reducing weight (53.1%). There was a high-level of adherence to HKCC social marketing messages and overall program structure, with few Local Project Manager reports of adaptations to theme one (9.2%) and theme two messages (15.4%). Fewer Local Project Managers (50%) reported the existence of private partnerships. While most respondents agreed they had the appropriate information to complete mandatory reporting, the usefulness of the HKCC online networking platform was in question (only 47% of Local Project Managers agreed that it was useful). Results reveal sufficient funding from the province to support program implementation, with a moderate level of local political commitment (63% of respondents).

**Conclusions:**

Results indicate that the HKCC was considered beneficial for enhancing access to health promoting programs, could be feasibly implemented with adherence to centrally-developed social marketing messages, and was amendable to local adaptation. Despite this, few private partnerships were reported. Going forward, there is opportunity to further evaluate factors contributing to HKCC program implementation, particularly as it relates to buy-in from intervention providers, and strategies for forming private sector partnerships to support long-term program sustainability.

## Background

In Canada, rates of overweight and obesity represent a significant public health challenge, having risen substantially since the 1980s [1]. Among children in 2017, 27.9% of 5–17 year olds are classified as overweight or obese, and obesity in childhood has been shown to track into adulthood, worsening in most individuals over time [2]. Prevalence trends are similar in England, where 30% of children (aged 2–15 years) were overweight (13%) or obese (17%) in 2017 [3]. Comparably, 18.5% of youth (aged 2–19 years) are obese in the United States [4], and in Australia, the prevalence of overweight and obesity in children (aged 4–18 years) is 16.4 and 7.0%, respectively [5]. In response to rising rates of overweight and obesity and related chronic diseases, community-based interventions have been designed to modify the risk factors associated with their development [6, 7]. With the community as a geographical setting, these interventions focus on changing the context for health behaviours among individuals in order to reduce the population-level risk of disease [8].

Drawing on the Ensemble Prévenons l’Obésité Des Enfants’ (EPODE, *Together Let’s Prevent Childhood Obesity*) model [9], the Ontario, Canada, Ministry of Health and Long-Term Care (MOHLTC) designed and funded the Healthy Kids Community Challenge (HKCC). The HKCC, a community-led, provincially-coordinated program, officially launched in September 2015, with 45 Ontario communities receiving funding to participate from the provincial government [10]. The objective of the (HKCC) is to reduce the prevalence of, and prevent child and youth overweight and obesity, through community-based initiatives to improve health behaviours in children. Participating communities work with local partners to develop and implement initiatives (programs, policies, environmental supports) that promote healthy lifestyles and are aligned with centrally developed social marketing themes and messages.

MOHLTC supports HKCC communities (i.e., Inputs) through the provision of funding, social marketing themes, training and tools, and other resources. A Scientific Reference Committee and Healthy Kids Resource Centres provide evidence-based advice and training, respectively. The HKCC has been designed to be consistent with the EPODE model, which includes social marketing, political commitment, sustainable resources, support services, and evidence [11] (see: Program Logic Model: Additional file 1).

EPODE was developed based on a successful model of community programming to address risk factors for childhood obesity in northern France [11]. EPODE programs include a cycle of campaigns, informed by evidence, local data and partnerships [11]. Previous studies evaluating EPODE interventions have demonstrated positive outcomes. For example, results of a campaign to promote water intake and reduce intake of sugar sweetened beverages (SSBs) indicate a reduction in average SSB consumption and average SSB servings for children in the intervention group as compared to the control group, after 1 year of intervention [12]. This study also found that the number of children bringing SSBs to school was lower in the intervention group. These findings imply that reduced SSB intake can have beneficial effects on total energy intake and weight status [13, 14].

Results from a study measuring rates of childhood (aged 5–12 years) obesity and overweight in two French towns [15] found a decrease in trends in mean body mass index (BMI) and prevalence of overweight between the years 1992 and 2004. Specifically, in the 2004 school year, overweight prevalence was significantly lower (8.8%) in the French intervention towns than in comparator towns (17.8%) [15]. Results of both van de Gaar et al. [12] and Romon et al. [16, 17] provide evidence of the value in the EPODE model for both improving health behaviours, and reducing overweight in children, over a long period of time.

Since the HKCC is a large, complex intervention, it requires an equivalently large and complex evaluation plan [18]. The MOHLTC requested that Public Health Ontario (PHO) assess the extent to which the HKCC objectives – to reduce the prevalence of, and prevent child and youth overweight and obesity – are achieved over a three-year intervention period (i.e., the HKCC timeframe) from 2015 through 2018. As part of this evaluation, an important component was an early assessment to describe and understand how the program was being implemented across the 39 municipal communities, and to identify opportunities for improvement [19]. A process evaluation can help identify potential negative outcomes or factors that may be contributing to adverse outcomes, and improve our understanding of positive outcomes [20].

Guided by Durlak and DuPre’s Ecological Framework for Understanding Effective Implementation [21], and considering the core components of the EPODE logic model, our evaluation focused on two objectives: 1) to describe the organization of the program at the community level; and, 2) to identify opportunities for improvement through an early assessment of factors contributing to implementation [9].

## Ecological framework

**Fig. 1:**
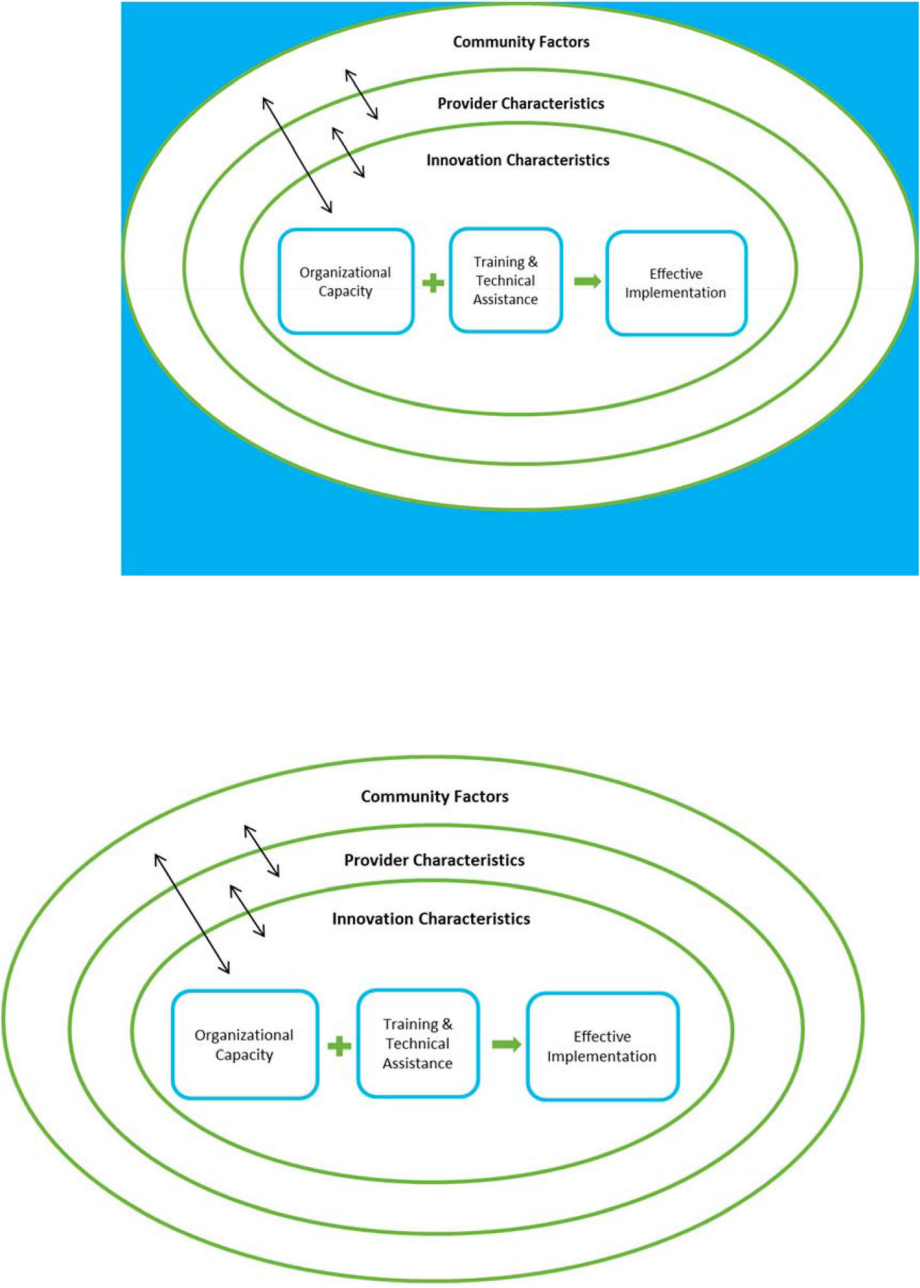
Durlak and DuPre’s Ecological Framework for Understanding Effective Implementation

Durlak and DuPre’s framework allowed for the assessment of HKCC program implementation relative to the levels and constructs of the framework to identify opportunities for improvement. The framework consists of five categories in which variables impacting implementation of community-based interventions are present [21], and is thus relevant to the HKCC, which includes community characteristics as key constructs. Second, the framework provides guidance on constructs at other levels that are relevant to the HKCC. The framework is presented in Fig. 1 as it applies to implementation of the Ontario HKCC program (with some adaptations) [21].

I. **Community level factors**: HKCC community context in which the program is implemented (politics, funding, policies, community demographics)
II. **Provider characteristics**: characteristics of the Local Project Managers and local steering committee members administering the program (perceived need for and benefits of the HKCC, self efficacy, self-proficiency)
III. **Characteristics of the innovation**: features that describe the HKCC program (compatibility, adaptability, evidence, integration of new programming)
IV. **Factors relevant to the prevention delivery system – organizational capacity**: extent to which the local steering committee and partner organizations can deliver the program (organizational factors, staffing considerations)
V. **Factors relevant to the prevention delivery system – training and technical assistance**: training (i.e., active training, ongoing resources) to effectively prepare providers (i.e., Local Project Managers, local steering committee member) to complete HKCC tasks

In addition to assessing participant perspectives on factors associated with implementation, we sought to collect information to describe the implementation of the HKCC as baseline for subsequent evaluation.

## Methods

### Program design

Within Ontario, a standardized local delivery model, prescribed social marketing themes, and detailed planning and reporting requirements help to ensure consistent implementation of the HKCC across communities, while allowing for local adaptation. The model requires that each community hire a Local Project Manager to lead partnership development, planning and program implementation, and MOHLTC reporting [16]. In the 39 municipal-HKCC communities that are the subject of this study (n.b., six Indigenous communities are being engaged in a separate evaluation), program funding flows from the Ministry to the Municipality.^1^ In some cases, a municipal department (e.g., Children’s Services) leads the HKCC by hiring a Local Project Manager to recruit multi-sectoral partners, and to establish a local steering committee to coordinate and plan the program. In other cases, the Municipality flows the funding to a separate organization leading the HKCC (e.g., Public Health Unit, local non-profit organization, etc.).

A centrally developed social marketing campaign was designed to motivate communities to organize interventions around pre-defined themes. Every 9 months, a new theme was announced to address factors associated with childhood overweight and obesity [17]. The first theme was *Run. Jump. Play. Every Day.* (1; October 2015–June 2016), followed by *Water Does Wonders* (2; July 2016–March 2017), *Choose to Boost Veggies and Fruit* (3; since April–December, 2017), and *Power Off and Play*! (January–September, 2018) [17].

Activities included central coordination and assistance to establish local steering committees, identification of community champions,^2^ conducting community needs assessments, and implementing theme-based action plans. For each theme, community initiatives (programs, policies, environmental supports), were informed by centrally developed planning tools, resources and scientific evidence designed to address local needs and build on local assets.

MOHLTC provided resources to support the development of interventions and local program implementation to Local Project Managers. MOHLTC provided communities with: funding, training and capacity building, evidence-based advice, Local Project Manager and community toolkits (e.g., communication tools and resources) to support a single health promotion theme [16]. Provincial HKCC supports were provided by: scientific and expert advice from the Scientific Reference Committee and an Aboriginal Stream Scientific Subcommittee (ASSSC), provincial evaluation support from PHO, social marketing support (through the Communications and Marketing Division, MOHLTC), and training and capacity building through the Healthy Kids Resource Centres.

### Study design

Data for this analysis were obtained from the first cycle of the local steering committee survey (see Additional file 2); a second survey cycle followed in Fall 2018. During both survey cycles, local steering committee interviews were undertaken in order to complement and/or verify findings from the local steering committee survey, and provide increased richness to results. The local steering committee survey utilized a repeat cross-sectional design; findings are presented from the first data collection time-point in December 2016. The survey was completed online by local steering committee members using SurveyMonkey. Quantitative measurement and qualitative inquiry are presented through data from fixed-choice (closed) and open-ended questions.

HKCC communities (*n* = 39) received the survey through a snowball sampling method, participation was voluntary, and participants could stop their participation at any time. Of the 785 local steering committee members who received the survey, 40% (*n* = 320) completed some component of the online survey.

### Measures

Guided by Durlak and DuPre’s Framework [21], survey questions (see Additional file 2) were developed specifically to enable evaluation of HKCC implementation based on constructs identified in the framework (see Section 4) that are known to be associated with implementation (e.g., program compatibility and adaptability, participant opinion on program benefits). Survey questions were informed by relevant literature [9, 11] and preliminary themes identified during the local steering committee interviews. The survey asked participants about their: role as it relates to the HKCC; attitudes and perceptions about the program; perceptions about program implementation (including barriers and facilitators); and suggestions for improvement. Participants were also asked to provide demographic information (age, gender, community, organization), and could respond to open-ended questions embedded within the quantitative survey questions. Individuals who identified as Local Project Managers were asked additional questions given their unique role.

The survey was pilot tested to test study procedures and validity of the survey tool with a small number of internal staff participants (*n* = 6) and external stakeholders (*n* = 7) from three communities selected purposively based on the fact that the research team had been in communication with them, and that there was a high likelihood that they would provide a response. Upon completion of the pilot, the survey was minimally adapted to improve clarity and the ease with which respondents could read and understand the survey questions.

### Data analysis

Data were checked for potentially duplicate participant responses. Each individual record was screened; records were deleted if there were no responses beyond the “general responses,” (e.g., role, community) and if responses to the general questions matched other records. Results include descriptive statistics of quantitative measures, analyzed using SAS EG 7.1. Due to the high frequency of positive responses, data were grouped into two binary categories, “Strongly Agree/Agree” and “Other.” The “Other” category includes “Neither agree nor disagree,” “Disagree/Strongly Disagree,” and “Don’t know” results.

Thematic analyses of a random sample (20%; *n* = 50) of qualitative responses to open-ended questions was undertaken to provide context and credibility to the quantitative data [22], at which point thematic saturation was reached. Results include theme counts with example quotations (see Additional file 3), which help to compliment, corroborate or refute, and further explain findings. Three steps were taken to improve the extent to which the qualitative data were analyzed in a rigorous way. First, a theme code set was developed and refined using constructs outlined in Durlak and DuPre’s Famework [21]. In order to test the theme code set and its application to the data, two team members undertook an inter-rater reliability exercise by coding three randomly selected transcripts, which resulted in a score of 75% [22]. Multiple coding helps to increase the rigour and trustworthiness of data, and involves cross checking of the coding strategy and interpretation of data by independent researchers [23]. According to Barbour [23], the degree of agreement – inter-rater reliability score – between coders is actually less important than the content of disagreements and insights resulting from discussion, which help to refine the coding strategy, and lead to alternative interpretations. Miles and Huberman indicate that an interrater reliability score near or above 80% is acceptable [24]. Therefore, evidence indicates this is an acceptable score. Second, codes were assigned to a sample of transcripts (*n* = 50; 20%) using NVivo to organize and code data. Third, thematic analysis of coded data was undertaken [25].

## Results

### Response rate and missing data

Participants were required to provide a response (each question included a “prefer not to answer” option) in order to advance through the online survey. Thus, missing data on closed-ended questions represents the end of participation. 82.8% (***n*** **= 265**) of participants completed the entire survey, while most (95.6%; ***n*** **= 306**) participants started it, completing at least 25% of the survey, including demographics. Given attrition as participants advanced through the survey, and that missing responses thereby constituted between 2.5 and 21.7% of the total responses depending on the item, percentage excluding missing is reported throughout.

To identify the number of eligible participants and to identify the denominator for categorical responses, Local Project Managers from every municipal HKCC community (*n* = 39) reported the number of individuals to whom they distributed the survey link.

### Participant characteristics

Of the participants who completed the survey, 25.9% (*n* = 83) self-identified as Local Project Managers, while 74.1% (*n* = 237) identified as non-Local Project Manager local steering committee members. Since the survey was conducted one-year after program launch, and 2 years after funding applications were submitted, communities may have hired more than one Local Project Manager over this time period or had multiple individuals fulfilling these responsibilities. All items asked of both Local Project Managers and non-Local Project Manager local steering committee members are grouped together (as local steering committee members). Survey questions that were unique to the roles are summarized separately. Thirty-nine per cent of participants represented an HKCC community champion.

All 39 municipal HKCC communities were represented; and, communities had between 1 and 24 individuals participate in the survey. Most Local Project Managers (LPM) and Local Steering Committee (LSC) members had been in their role for between 13 and 24 months (56.8% versus 49.5%, respectively) or less than 1 year (28.4% versus 41.1%, respectively). Local Project Managers (*n* = 83) were hosted by: a local municipality (*n* = 27); the recreation sector (*n* = 20); the non-profit sector (*n* = 19); and/or a public health unit (*n* = 16). Local steering committee members (*n* = 237) were represented by: the non-profit sector (*n* = 50); a local municipality (*n* = 49); as being a local community member (*n* = 45), and/or a local public health unit (*n* = 40).

Nearly half (45.6%) of non-Local Project Manager local steering committee members were actively involved in the functioning of the HKCC partnership, while 24.5% provided some form of specific support to the project. Table 1 provides an overview of tasks undertaken, where the most frequently reported tasks include: a) planning HKCC initiatives in your community (*n* = 166); b) sharing knowledge and expertise (e.g., data or information on social marketing expertise, etc.) (*n* = 164); c) mobilizing and encouraging your community to be involved in the HKCC (*n* = 154); and, d) developing theme-based action plans (*n* = 143).

**Table 1:** Most frequently reported tasks undertaken by local steering committee members (including Local Project Managers) for the first HKCC theme

What are the three tasks that you spent the most time on as a local steering committee member for the first HKCC theme? (please pick three only) (*n* = 320)	n
a. Planning HKCC initiatives in your community	166
b. Sharing knowledge and expertise (e.g., data or information on consumer behaviours, social marketing expertise)	164
c. Mobilizing and encouraging your community to be involved in the HKCC	154
d. Developing Theme-Based Action Plans	143
e. Participating in local HKCC events in your community	88
f. Providing input into evaluation and data collection	57
g. Planning or conducting community needs assessment	47
h. Negotiating private or public partnerships	32

Participants could select up to three responses

The remainder of results have been organized and mapped onto the five categories outlined by Durlak and DuPre’s Framework [21].

#### Community level factors

82.4% of participants agreed that there was strong support from local community partners for HKCC implementation, while 63.7% agreed there was sufficient funding from the province to support program implementation. In addition to fairly strong local political commitment (63%), half (51%) of respondents reported that there were sufficient existing policies to support implementation. Only 24.2% of Local Project Managers indicated that their community had received extra financial support for HKCC implementation (see Table 2), while nearly 65% of participants reported that their organization had provided in-kind support for HKCC functioning. These findings are corroborated by the qualitative data (see Additional file 3), in which respondents identified the important role of funding (*n* = 79 mentions: in-kind, funding for programming, activities, sustainability, evaluation). For example, this support included “*Free use of municipal grounds, storage space and community centre, free printing and supplies, volunteer hours for equipment assembly and installation, staff time for equipment purchasing and lending hub set up*”.

**Table 2:** Perceptions of financial/in-kind support for the HKCC program at the community-level

	Yes	No	Don’t know/ Prefer not to answer
a. Did your community receive extra financial support for HKCC implementation in your community (Local Project Manager responses; *n* = 83)?	24.2	34.8	42.5
b. Did your organization provide in-kind support for HKCC functions (non-Local Project Manager local steering committee responses; *n* = 237)?	64.3	16.2	20.0
c. Did your organization contribute additional funding to support HKCC activities (e.g., planning, evaluation) (local steering committee member responses; *n* = 320)?	36.7	41.0	22.3

Missing responses constituted between 2.5 and 21.7% of the total responses; percentage excluding missing is reported

#### Provider characteristics

Most participants (93%) agreed that the program was beneficial to their community, while 88.3% reported that it enhanced individuals’ access to community programs and activities (Table 3). A high proportion (83.1%) of respondents agreed that the program increases knowledge of health behaviours in the community, while 63.8% believe that it actually changes health behaviours. Despite this, fewer respondents (53.1%) agreed that the HKCC is effective in reducing childhood overweight and obesity in their community.

**Table 3:** Local steering committee members’ (including Local Project Managers) perception of needs and benefits of the HKCC/organizational characteristics

Local steering committee members’ (including Local Prooject Managers) perception of needs and benefits of the HKCC.			
Please indicate the extent to which you agree or disagree with each of the following statements^a^ (*n* = 320)	Yes	Other	Prefer not to answer/Missing
a. The HKCC program benefits my community	93.1	6.6	0.3
b. The program enhances access to programs and activities in the community	88.3	11.4	0.3
c. The HKCC is effective in increasing knowledge of health behaviours in my community	83.1	16.2	0.7
d. HKCC contributes to a sense of community	77.6	22.4	0
e. I believe it is effective in changing health behaviours in my community	63.8	34.5	0.7
f. I believe the HKCC is effective in reducing childhood OW/O in my community	53.1	46.2	0.7
Local steering committee members’ perceptions of organizational characteristics.			
Please respond to the following statements (*n* = 269)	Yes	Other	Prefer not to answer/Not applicable
a. The local steering committee has collaborated effectively to facilitate HKCC implementation	89.2	8.9	1.8
b. There is strong leadership on the local steering committee	86.3	11.9	1.9
c. There is trust among members of the local steering committee	84.4	11.9	3.7
d. The local steering committee has a process for decision-making	80.4	14.8	4.9
e. Community champion(s) have supported the implementation of HKCC activities in our community	77.0	18.6	4.5
f. Sub-committees from the local steering committee were helpful in planning and implementing HKCC related activities	66.9	17.5	15.6

Missing responses constituted between 2.5 and 21.7% of the total responses; percentage excluding missing is reported

^a^Yes = Strongly Agree/Agree; Other = Neither Agree Nor Disagree, Disagree or Strongly Disagree)

Qualitative results are mostly consistent with quantitative results, including a high level of agreement about perceived benefits of the HKCC (*n* = 39 mentions related to: community outcomes, knowledge or awareness of HKCC health behaviours, health behaviour changes) (see Additional file 3). For example, “*It has also been great to see the amount of media attention that HKCC has attracted and the ‘buzz’ it is generating. Kids and their families are recognizing our HKCC branding and they are excited about our activities*”.

Overall, most local steering committee member participants (including Local Project Managers) were confident that they: had the skills needed to engage with community partners (93.5%); could achieve the goals of the HKCC in their community (85.5%); and, could implement the HKCC in the community (81.4%). Some (59.7%) were less confident and found it challenging to engage community partners to participate in the HKCC. As one participant articulated in the qualitative data: “*Our committee has good representation/participation from those with local expertise in community development, program planning and evaluation.*”

#### Characteristics of the innovation

Participants largely agreed that the HKCC program was compatible, adaptable, and acceptable to their communities. For example, 89.1% (*n* = 277) of participants agreed that the HKCC could be adapted to fit local needs, while 86.8% agreed that initiatives were feasible to deliver. A majority (79.4%) of respondents perceived that there was general public support for the HKCC, while 71.4% perceived that social marketing campaigns were well-received. Although respondents indicated that the program was compatible with and adaptable to their communities, few (13.8%; *n* = 9) Local Project Managers reported that the actual HKCC brand had been adapted. While 9.2% of participants reported that messaging adaptations had occurred for *theme one* (Run. Jump. Play. Every Day.), 15.4% indicated that their community had adapted the messaging for *theme two* (Water Does Wonders). These findings are noteworthy in that adherence to specific HKCC brand and theme messaging may be important to implementation fidelity. A majority of respondents provided qualitative data (*n* = 61) data related to the adaptability of the HKCC (e.g., to reach vulnerable populations) (see Additional file 3). For example, one respondent revealed: “*Within my community we have implemented a program that directly targets at-risk and marginalized youth by offering them leadership training and a substantive voice at the planning table*”.

Participants were asked to report on the use of evidence to inform the development of the HKCC program. As such, 86.6% (*n* = 259) of participants (including Local Project Managers) agreed that *theme two* “Water Does Wonders” was based on sound scientific evidence, while the same was true (86.0% agreement) for *theme one* “Run. Jump. Play. Every Day.” There was a high level of agreement (86.3%) for a strong evidence base supporting the provincial HKCC program. Further, nearly 70% of respondents agreed that *systematic methods* had been used to search for evidence to inform local HKCC activities and programs.

#### Prevention delivery system – organizational capacity

Results indicate that a high proportion (89.2%) of participants believed that local steering committee collaborations helped to facilitate program implementation, while 86.3% recognized strong leadership for the local steering committee (see Table 3). Trust among local steering committee members and strong decision-making processes were observed by 84.4 and 80.4% of participants, respectively. Qualitative results reflect quanitative results, with 97 mentions of mostly positive group relationships between stakeholders (e.g., group relationships, collaboration, commitment, participation). Collaboration was viewed as an important theme, particularly in the context of organizational capacity to deliver the HKCC program,

*We have seen community organizations with similar programs/initiatives willing to come together to discuss how they can, with some compromises, work together to strengthen their programs/services objectives. We have seen some creative projects arise and have become stronger through collaborative evolution, via the idea generating forums we host around each HKCC theme as it is revealed*.

Half of Local Project Managers (50%; *n* = 34) reported that they have developed informal private partners (without a partnership charter) on the HKCC, while fewer (11.8%; *n* = 8) have formal private partnerships. More than 32% (*n* = 22) of respondents reported that their HKCC community did not have any private HKCC-related partners.

Eighty-three per cent of participants agreed that partners shared a vision and goals related to the HKCC (see Table 4). There was a high level of agreement (78.2%) about the extent to which effective communication channels among partners had formed, in addition to improved coordination (75.7%). There was also agreement (73.1%) that partnerships are likely to sustain beyond the HKCC program end.

**Table 4:** Local steering committee member perceptions of benefits of community partnerships (reported by local steering committee members)/Local Project Manager perceptions of training and technical assistance

Local steering committee member perceptions of benefits of community partnerships (reported by local steering committee members)			
Please indicate the extent to which you agree with the following statements^a^(*n* = 271)	Yes	Other	Don’t know/ Prefer not to answer
a. There is a shared HKCC-related vision and shared goals among partners	83.4	16.2	0.4
b. Effective communication channels among partners have formed	78.2	21.4	0.4
c. Coordination among partners has improved	75.7	23.6	0.7
d. Partnerships formed from the HKCC are likely to continue after HKCC funding ends	73.1	26.6	0.4
e. Trust among partners has increased	68.3	31.4	0.4
f. Collaboration on spin-off projects has increased	59.8	39.9	0.4
Local Project Manager perceptions of training and technical assistance.			
Please indicate the extent to which you agree with the following statements^a^(*n* = 99)	Yes	Other	Don’t know/ Prefer not to answer
a. There are sufficient resources and support to implement the HKCC in my community	80.6	15.3	4.2
b. The resources/materials provided by Healthy Kids Resources Centres are useful	71.6	23.0	5.4
c. The Local Project Manager guidance document clearly defines my roles and responsibilities	68.9	21.6	9.5
d. I have the information I need to complete MOHLTC reporting requirements	65.3	19.4	15.3
e. 1:1 support from MOHLTC has addressed my specific questions or concerns	58.1	15.7	21.6
f. Local Project Manager in-person training was useful	56.8	25.7	17.6
g. The online networking platform is useful for sharing with and learning from other Local Project Managers	47.3	36.5	16.2
h. Healthy Kids Resource Centre 1:1 support has addressed my specific questions or concerns	45.9	24.3	29.7

Missing responses constituted between 2.5 and 21.7% of the total responses; percentage excluding missing is reported

^a^Yes = Strongly Agree/Agree; Other = Neither Agree Nor Disagree, Disagree or Strongly Disagree)

There was a high level of agreement with respect to community structures and partnerships. For example, 93% of respondents indicated that their local steering committee developed linkages with community groups and organizations to spread HKCC messages in the community and to expand their own programs to include HKCC initiatives (86%). Most (91.5%) respondents agreed that their local steering committee had developed links with pre-existing community structures, while 81.9% agreed their local steering committee is or had networked with diverse sectors to gain support for the HKCC program.

#### Prevention delivery system – training and technical assistance

Results reveal fairly strong respondent agreement that there are sufficient resources and support to implement the program (80.6%), and that resources and supports offered by the Healthy Kids Resource Centres are useful (71.6%). More than half of respondents indicated that 1:1 support from MOHLTC helped to address specific questions or concerns (58.1%), and that Local Project Manager in-person training was useful.

## Discussion

Guided by Durlak and DuPre’s Ecological Framework for Understanding Effective Implementation [21] and considering the core components of the HKCC logic model (Additional file 3), the objectives of our evaluation were: 1) to describe organization of the HKCC program at the community level; and, 2) to identify opportunities for improvement through an early assessment of factors contributing to program implementation.

Perceived program need and benefit are provider-level factors that contribute to implementation success [26]. While respondents were generally positive about the program and agreed that the HKCC was beneficial, there was less agreement that the program could reduce childhood obesity. We note that HKCC social marketing materials do not include specific messaging about childhood obesity, and instead focus on healthy behaviours and healthy kids. In addition, participants may have felt that a three-year population health intervention was too short to impact rates of overweight and obesity. As such, survey findings may represent an appropriate response to program duration and MOHLTC messaging about the program.

Feasibility to implement with fidelity is essential to achieve program outcomes [27]. HKCC program fidelity is expressed, in part, through a high level of adherence to the centrally-developed social marketing messages delivered around specific health promotion themes. However, the success of community-based initiatives also requires that fidelity is balanced by local program adaptations based on local context [28, 29]. Other social marketing interventions in public health have been evaluated for fidelity. For example, results from the VERB Campaign – a mass social marketing campaign targeting children aged 9–13 years to increase levels of physical activity – indicate a high level of awareness and understanding of the campaign, in addition to a positive impact on rates of physical activity [30].

The presence of a shared vision and goals, and strong communication channels among partners within HKCC communities, are critical attributes of successful program planning and implementation [31]. Sustainability of partnerships is a key component of population health interventions that intend to address long-term, complex issues, leading to interventions that are more likely to maintain beyond a funding period [32].

Community partnerships are integral to spreading key messages about the program, and for leveraging resources – in-kind (i.e., person hours), material (i.e., sports equipment), and financial, and have been identified as a key success factor of EPODE-modelled interventions world-wide [11]. Partnerships add capacity and infrastructure, and are essential to sustaining momentum after the program funding ends. Few private partnerships were reported, which may represent a lack of experience in working with the private sector, or unaddressed concerns about engaging private sector in health promotion activities [33].

Mobilizing stakeholders at all levels to shape and change obesogenic environments requires the active support of program developers, implementers, evaluators, and policymakers [34]. Private sector partnerships require explicit guidelines to help facilitate partner relationships between industry, government, non-profit agencies, and other related stakeholders [35]. Guidelines include: items related to building trust, information sharing, transparency, resource pooling, communication channels, and appropriate leadership [35]. Our findings support the need for explicit guideline and policy development for partnership formation. Canada’s ParticipACTION Partnership Protocol (2010) could be a useful starting point to these activities (see: http://www.mindingourbodies.ca/sites/default/files/partnershipprotocol_english_final.pdf).

Funding and political support are deemed essential for effective implementation [11]. Results reveal sufficient funding from the province to support implementation, and a moderate amount of local political commitment. However, few existing policies were reported at the local level to support program implementation. This finding was not surprising, since policy development takes time and the focus of this survey was on the first of a three-year (2015–2018, inclusive) intervention. Subsequent evaluation activities assess the extent to which the program itself might stimulate the development of policies to support health behaviours in children and families.

Brownson [36] reiterates the important role of evidence, informing all judgements about policy, programs and system change. These, and other findings highlight the need to articulate the user group for which evidence is produced, including public health practitioners, public and private partners, policymakers at local/regional, provincial, national and international levels, non-governmental stakeholders (e.g., public interest groups, individuals), and population health intervention researchers [36, 37].

Comparing our results with other programs, Pettigrew et al. [38] identified the following issues associated with 25 existing EPODE-modelled programs: the inability of programs to secure long-term funding, and their needs for access to evidence-based intervention methods as it relates to relationships with private sector partners. The authors highlighted three main barriers to implementation and opportunities for improvement: assistance with developing and sustaining stakeholder relationships, access to user-friendly information related to interventions and evaluation, and confirmation of quality and transparency of policies and practices [38].

## Implications for future research and evaluation

Planning for future HKCC evaluation activities might include an in-depth assessment of the role of external stakeholders, including individuals from the private sector, who are actively supporting program implementation. These partners may have a less formal – but equally important – role in the functioning of the program, particularly in the context of long-term program sustainability. For example, strong partnership functioning with stakeholders from public-and private-sectors can facilitate effective and efficient program development, delivery, and maintenance [39]. Evaluation is critical for sustaining viable partnerships, including to assess partnership infrastructure, function, and processes, noting that the needs and measures associated with evaluation may vary depending on the type of partner (i.e., private or public) [39].

Implications for future research include the need to further explore functioning of the local steering committee through case study methodology. As per recommendations provided by Moore and others [40], case study research can help elucidate causal mechanisms impacting program implementation. Adopting a realist case study evaluation approach [41, 42] would further our understanding of theories of change embedded within the HKCC program. In doing so, findings could help to improve and strengthen the way in which the HKCC is both implemented and sustained long-term, including a contribution to the international literature.

Our study contributes a case to the growing literature on implementation of community-based initiatives. Our evaluation used a framework to assess early implementation that was deemed relevant to the HKCC program as it includes multi-level factors impacting implementation of community-based interventions, and included community factors as key constructs [21]. Other frameworks could be used to reflect other innovation characteristics, such as the complexity of the intervention. For example, Caroll and others [43] developed a conceptual framework to assess elements and relationships between adherence and moderators of implementation fidelity that includes intervention complexity (i.e., detailed or specific), facilitation strategies (e.g., provision of manuals, guidelines, training, and monitoring and feedback for program deliverers), quality of delivery (i.e., whether the intervention is delivered in an appropriate way to achieve the goals), and participant responsiveness (i.e., acceptability of the intervention by those who are receiving it) [43]. There is a need to build evidence on the strengths and limitations of various implementation frameworks, and guidance required on how to select and apply the most appropriate framework for innovations implemented based on different settings, with different end-users, and with varying degrees of complexity [44, 45].

## Limitations

It is important to consider some limitations of this study. First, recruitment through snowball sampling was used to increase the potential participation rates. Although Local Project Managers were provided with clear instructions to forward the survey link to their entire local steering committee based on this method, we are not certain if there was equal distribution of the survey link across local steering committees or equal participation rates across the communities, and therefore results may not be representative of all local steering committee members or communities. This is not too problematic, since we do not claim that our results are generalizable to all communities or across all LSC members (including Local Project Managers). Also, although Local Project Managers indicated that they had successfully distributed the survey link to their local steering committee members, it is possible that the link was sent beyond the local steering committee, or that local steering committee members chose not to participate. It is also possible that not all members of the LSC received the survey, either because they were no longer involved in the HKCC at the time of the survey, or because they had missed the email invitation. Because of this method of recruitment, we could not track the number of times the survey link was distributed, and there was potential for respondents to participate in the survey more than once. Extensive efforts were made to identify and remove duplicates, but some may not have been detected and therefore their opinions may have been counted twice.

Second, this study may have experienced some selection bias, with more polarized Local Project Manager and local steering committee members more likely to participate in order to share their opinions. We did note, for example, that most responses were skewed positively, and will address this by combining response options for subsequent surveys. Also, we could not be entirely sure if Local Project Managers or local steering committee members reported their roles accurately, as evidenced by the number of survey respondents that self-identified as Local Project Managers. This point is further complicated by the fact that communities had between one and 24 individuals participate in the survey. Finally, as noted throughout the manuscript, the proportion of missing data increased as the survey progressed, which may be attributable to survey fatigue. This can be remedied in future iterations of the survey with a function added whereby participants can exit and return to the survey where they left off.

Given our selection and use of Durlak and DuPre’s [21] framework for effective implementation of community programs, we did not examine program participant or consumer factors impacting the implementation process. However, sociodemographic factors such as low income or geographic isolation that might impact on community member participation were captured by each community as part of their community needs assessment process, with the intention to address or mitigate these in local programming. Future studies are planned that will assess program reach across communities to understand the impact of sociodemographic factors on participation.

## Conclusion

Results indicate that the HKCC was considered beneficial for enhancing access to health promoting programs, could be feasibly implemented with adherence to centrally-developed social marketing messages, and was amendable to local adaptation. Despite this, few private partnerships were reported, and there was less agreement that the program could be beneficial in reducing or preventing childhood overweight and obesity over a 3 year period. Durlak and DuPre’s Ecological Framework for Understanding Effective Implementation [21] was shown to be a useful framework for assessing HKCC program implementation relative to the levels and constructs of the framework to identify early opportunities for improvement. Future research to collect implementation data is essential, specifically to better elucidate the factors that influence implementation within and between community settings.

## Supplementary information

**Additional file 1.** Ontario’s Healthy Kids Community Challenge Program Logic Model.

**Additional file 2.** HKCC Local Steering Committee Survey.

**Additional file 3.** Summary of qualitative results, including themes, number of mentions and selected quotations.

## Data Availability

The datasets used and/or analysed during the current study are available from the corresponding author on reasonable request.

## Abbreviations

EPODE model: Ensemble Prévenons l’Obésité Des Enfants’, *Together Let’s Prevent Childhood Obesity* model
HKCC: Healthy Kids Community Challenge
MOHLTC: Ministry of Health and Long-Term Care

## Footnotes

1 “A municipality is defined in section 1 of the Municipal Act, 2011 as a “geographic area whose inhabitants are incorporated.” Section 2 of the act provides that “municipalities are created by the Province of Ontario to be responsible and accountable governments with respect to matters within their jurisdiction, and each municipality is given powers and duties under the act and many other acts for the purpose of providing good government with respect to those matters”” (Ontario Ministry of Municipal Affairs, Ministry of Housing, 2011, Section 2: An Overview of Local Government. Available at: http://www.mah.gov.on.ca/Page8391.aspx).

2 Community champion is defined as: “a leader who will support and promote the initiatives and activities developed and implemented. The Champion’s role could take a variety of forms but include activities that promote community partner engagements and raising awareness of the initiatives in the communities” ([17], p.10).
