# Supplementary figures and images for: An early implementation assessment of Ontario’s Healthy Kids Community Challenge: results from a survey of key stakeholders
Source: BMC Public Health. 2019 Nov 27;19:1568. doi: 10.1186/s12889-019-7704-2 (PMC6880511; doi:10.1186/s12889-019-7704-2)

**Additional File 1:** Ontario’s Healthy Kids Community Challenge Program Logic Model


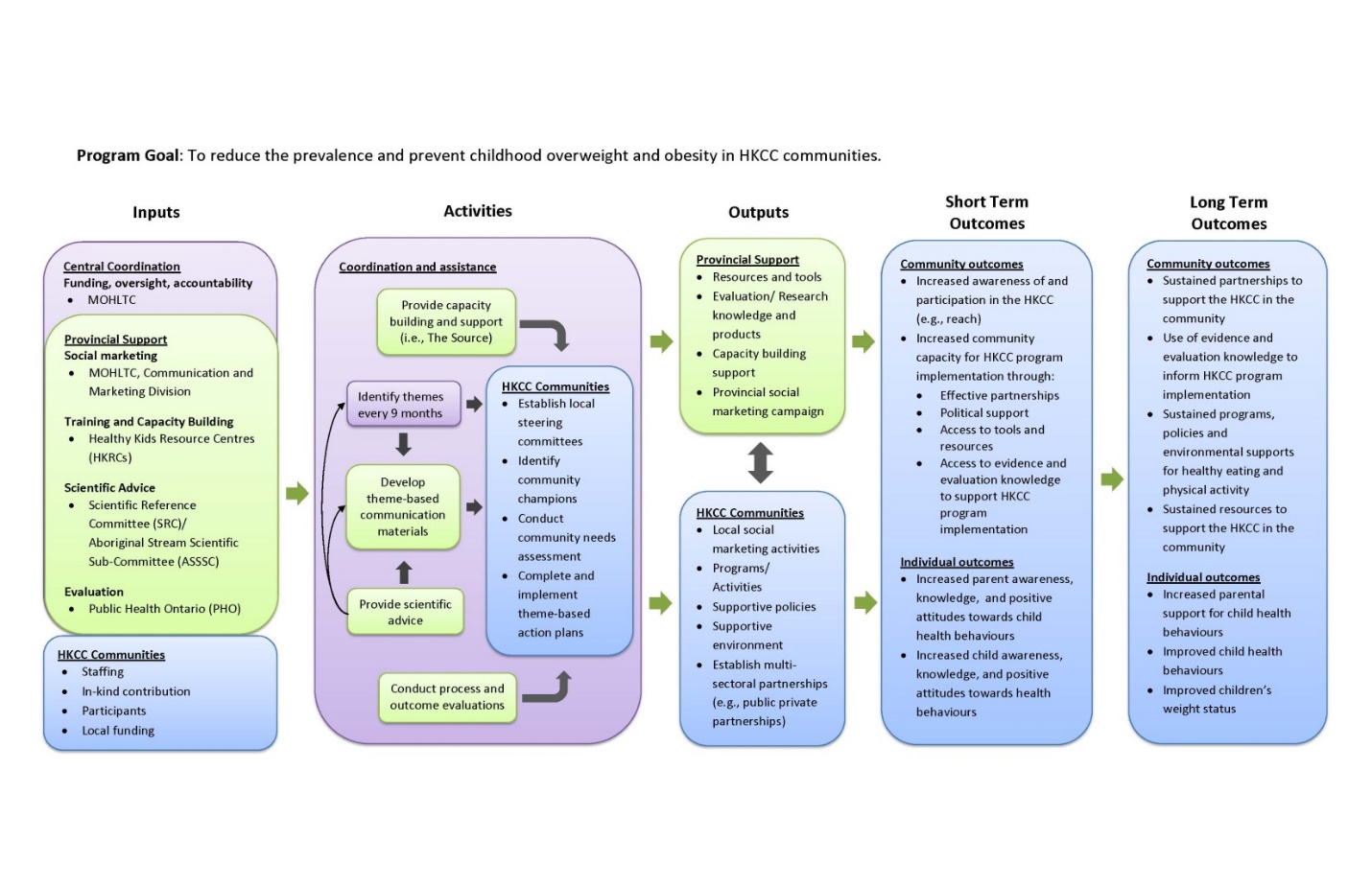

Supplement: Supplementary file 1 — Additional file 1. Ontario’s Healthy Kids Community Challenge Program Logic Model. [file 12889_2019_7704_MOESM1_ESM.docx]
