# Supplementary material for: An early implementation assessment of Ontario’s Healthy Kids Community Challenge: results from a survey of key stakeholders
Source: BMC Public Health. 2019 Nov 27;19:1568. doi: 10.1186/s12889-019-7704-2 (PMC6880511; doi:10.1186/s12889-019-7704-2)
Supplement: Supplementary file 2 — Additional file 2. HKCC Local Steering Committee Survey. [file 12889_2019_7704_MOESM2_ESM.pdf]

Introduction:
You are invited to take part in a survey about your role as **a <u>local project manager or a member of the local steering committee</u>** with the Healthy Kids Community Challenge (HKCC). The survey is part of a larger evaluation being conducted by Dr. Heather Manson and the HKCC Evaluation Team at Public Health Ontario. Public Health Ontario (PHO) is a Crown corporation and an arm's length government agency dedicated to protecting and promoting the health of all Ontarians and reducing inequities in health. To learn more about PHO, please visit http://www.publichealthontario.ca/en/About/Pages/Organization.aspx.

Before agreeing to take part in this survey, it is important that you understand its purpose and what is involved. This information sheet includes important details that will help you decide if you wish to take part in the survey.

Evaluation Sponsor:
This evaluation is being funded by Public Health Ontario. PHO acknowledges funding from the Ministry of Health and Long-Term Care (MOHLTC).

Purpose of the Evaluation:
The purpose of this survey is to understand the factors that may contribute to HKCC program implementation in your community. As a local project manager or local steering committee member, your perspective is important to us to learn more about how community partners can work together to improve community health and to understand how the HKCC has been implemented.

Procedures:
The online survey asks about your role in and attitudes towards the HKCC, its benefits to your community, support for the HKCC, program implementation (including barriers and facilitators), and suggestions for improvement. We will also ask you to provide some basic demographic information (e.g., age, gender, community, organization). **The survey will take about 30 minutes to complete.**

Potential Benefits:
The survey will not benefit you directly; however, we hope to learn more about your experiences implementing the HKCC by better understanding stakeholder involvement, perspectives, and role in the HKCC at the community level. Findings may help to inform local and provincial level policy (e.g., school nutrition/physical activity), and land use planning (e.g., built environment: walkability, public park design, bike lanes).

Potential Risks and Burdens:
The risks involved in participating in this survey are low. For example, there are questions about your own experience related to the program implementation that you might feel uncomfortable answering. <u>Aside from indicating which community you are representing and your role</u>, you are free not to participate, to skip a question or to end your participation at any time with no consequences to you. You can withdraw (stop taking part) from the survey at any time.

Privacy/Confidentiality:

This information is collected under the authority of the Ontario Agency for Health Protection and Promotion Act. Your participation in this survey is voluntary and by completing the questionnaire you consent to the collection of information. You can refuse to participate, or withdraw from the survey at any time. Data collected up to the point when you withdrawal will be retained by PHO. Your information will only be disclosed as permitted or required by law.

Please be aware that information in connection with your response to the survey will be stored on PHO servers throughout the data lifecycle (e.g., collection process, use for analysis, retention), and is governed by PHO Terms of Use. Access to data stored on PHO servers will be limited to online survey administrators and to PHO staff involved in this initiative. Data will be retained by PHO per PHO's Operational Records Retention Schedule [OAHPP-7-1R] before they are permanently deleted.

Where appropriate, responses will be analyzed at a community level. However, all participants will be de-identified before analysis. No communities will be named in any publications arising from this evaluation (e.g., reports, conference presentations, peer review publications). You will not be asked to provide your name in the survey, and it will not be possible for the PHO evaluation team to identify responses from specific individuals. Therefore, identifiable data will not be shared with the Ministry of Health and Long-Term Care (MOHLTC).

If you have questions about the project please contact the evaluation specialist, Rachel Laxer, at HKCCevaluation@oahpp.ca (647-260-7654).

This project has been reviewed by the PHO Ethics Review Board. If you have questions about your rights as a participant, or the PHO ethics review process please contact Tim Westberg, Research Ethics Coordinator, at 647-260-7206 or tim.westberg@oahpp.ca.

By checking the box, you are consenting to participate in the local project manager/local steering committee survey.

□ Yes, I consent to participate in the local project manager/local steering committee survey.

□ No, I do not consent to participate in the local project manager/local steering committee survey.

SURVEY

General Questions

The following questions help us understand the general characteristics of local steering committee members. **It will not be used to identify individual responses**.

1. In order to group responses by community, please indicate the community you are from **(NOTE: THIS IS A MANDATORY QUESTION)**:

• Alfred-Plantagenet & Bourget	• Town of Marathon
• Town of Ajax	• County of Middlesex
• The Town of Aurora	• Regional Municipality of Niagara
• Brantford-Brant	• City of Oshawa
• City of Burlington	• City of Ottawa
• Municipality of Chatham-Kent	• City of Peterborough
• Town of Collingwood	• County of Renfrew
• Town of Georgina	• City of Sault Ste. Marie
• South East Grey	• St. Thomas - Elgin
• City of Guelph	• City of Greater Sudbury
• City of Hamilton	• City of Temiskaming Shores
• Hastings and Prince Edward	• North Channel
• The County of Huron	• City of Thunder Bay
• Town of Kapuskasing	• City of Toronto - Danforth-East York
• City of Kenora	• City of Toronto - Humber-Downsview
• KFL&A Region (The City of Kingston)	• City of Toronto - Rexdale
• Lambton – Partners	• City of Toronto - Central Scarborough
• United Counties of Leeds & Grenville	• Township of Uxbridge
• City of London	• Regional Municipality of Waterloo
	• Windsor – Essex

2. **Are you the local project manager in your community? (Mandatory question):**
    a. Yes
    b. No

3. **Are you identified as a HKCC Community Champion?**
    a. Yes
    b. No

(FOR LPM)

4. How long have you been working as a local project manager in your community (in Years and MONTHS)? ____________________________________

5. Please indicate the organization in which you are being hosted as an LPM (your primary affiliation): (Formulation of tasks)
    a. Community members (parents/caregivers, other)
    b. Local municipality
    c. Local First Nation, or other Indigenous organization
    d. Public health sector
    e. Education sector
    f. Recreation sector
    g. Health care sector
    h. Non-profit sector
    i. Volunteer sector
    j. Business sector
    k. Other sector, please specify: _______________________________________
    l. Prefer not to answer

6. What are the three tasks that you spent the most time on as a local project manager? (please pick THREE only) (Formulation of tasks)
    a. Developing Themed-Based Action Plans
    b. Planning or conducting community needs assessment
    c. Negotiating private or public partnerships
    d. Sharing knowledge and expertise (e.g., data or information on consumer behaviours, social marketing expertise)
    e. Mobilizing and encouraging your community to be involved in the HKCC
    f. Planning HKCC initiatives in your community
    g. Participating in local HKCC events in your community
    h. Providing input into evaluation and data collection
    i. Prefer not to answer
    j. Other (please specify)

7. Which themes have you been involved with, in your role as LPM?
    a. Run. Jump. Play. Every Day.
    b. Water Does Wonders
    c. Choose to Boost Veggies and Fruit
    d. Power Off and Play

(FOR LSC)

Your community has established a **HKCC Local Steering Committee (LSC)** to coordinate planning and implementation of The Challenge (e.g., theme-based action plan, negotiating private partnerships, providing input on evaluation and data collection).

2. **How long have you been a member of your local steering committee (in Years and months)?**
   ______________________________________ (Duration of local steering committee)

3. **Please indicate the organization that best describes your role:**

   a. Community members (parents/caregivers, other)
   b. Local municipality
   c. Local First Nation, or other Indigenous organization
   d. Public health sector
   e. Education sector
   f. Recreation sector
   g. Health care sector
   h. Non-profit sector
   i. Volunteer sector
   j. Business sector
   k. Other sector, please specify: ____
   l. Prefer not to answer

4. **Please select the statement that most closely reflects your involvement**: (Formulation of tasks)
   a. You are actively involved in the functioning of the partnership.
   b. You are frequently consulted or part of the process.
   c. You provide some form of specific support to the project, such as participating in a function of the partnership or on a specific task force of the partnership.
   d. You are kept informed of the progress and work of the partnership, but are not directly involved in its work.

5. **What are the three tasks that you spent the most time on as a local steering committee member (please pick THREE only)** (Formulation of tasks)
   a. Developing Themed-Based Action Plans
   b. Planning or conducting community needs assessment
   c. Negotiating private or public partnerships
   d. Sharing knowledge and expertise (e.g., data or information on consumer behaviours, social marketing expertise)
   e. Mobilizing and encouraging your community to be involved in the HKCC
   f. Planning HKCC initiatives in your community
   g. Participating in local HKCC events in your community
   h. Providing input into evaluation and data collection
   i. Prefer not to answer
   j. Other (please specify)

INNOVATION CHARACTERISTICS

Compatibility and adaptability

6. **Please indicate the extent to which you agree or disagree with each of the following statements as they pertain to your community:**

	Strongly Agree	Agree	Neither agree nor disagree	Disagree	Strongly Disagree	Don't Know	Prefer not to answer
HKCC can be adapted to fit the needs in my community (adaptability, innovation)	o	o	o	o	o	o	o
HKCC initiatives are feasible for community partners to deliver (adaptability, innovation)	o	o	o	o	o	o	o
Key messages from the social marketing campaign(s) were positively received by the general public in my community (fit)	o	o	o	o	o	o	o

Health Equity

Some HKCC communities may choose to address vulnerable populations by targeting HKCC resources to improve health equity.

7. **Please indicate the extent to which you either agree or disagree with each of the following statements:**

	Strongly Agree	Agree	Neither agree nor disagree	Disagree	Strongly Disagree	Don't Know	Prefer not to answer
It is difficult to reach vulnerable populations to							

participate in HKCC initiatives in my community (reach, based on pilot comments)	o	o	o	o	o	o	o
We made an effort to reach vulnerable populations in the community (reach, based on pilot comments)	o	o	o	o	o	o	o

8. **Please describe the types of barriers (if any) that your community faced when trying to reach vulnerable populations**

____
____
____

9. **What strategies has your community implemented to increase participation of vulnerable populations in HKCC initiatives? Please describe them below:**

____
____
____

<u>Evidence</u>

10. **Please indicate the extent to which you agree or disagree with each of the following statements:**

	**Strongly Agree**	**Agree**	**Neither agree nor disagree**	**Disagree**	**Strongly Disagree**	**Don't Know**	**Prefer not to answer**
	o	o	o	o	o	o	o
The health benefits and risks of the HKCC population in my community were considered to inform our HKCC activities/programs (evidence, innovation)	o	o	o	o	o	o	o
Themes were based on sound scientific evidence (prevention theory and research)	o	o	o	o	o	o	o

In my opinion, evidence supporting the broad (i.e., provincial) HKCC program is strong (prevention theory and research)	o	o	o	o	o	o	o
In my opinion, the evidence supporting my community's HKCC initiatives is strong (evidence, innovation)	o	o	o	o	o	o	o

11. **Did you consult external scientists regarding program planning, implementation and/or evaluation?**

    a. **Yes/No/Unsure**

PROVIDER LEVEL

<u>Perceived needs and benefits</u>

12. **Please indicate the extent to which you agree or disagree with each of the following statements as they relate to your community:**

**In my community…**	**Strongly Agree**	**Agree**	**Neither agree nor disagree**	**Disagree**	**Strongly Disagree**	**Don't know**	**Prefer not to answer**
The HKCC program is beneficial (perceived benefits, provider)	o	o	o	o	o	o	o
There is a need for HKCC initiatives (perceived need, provider)	o	o	o	o	o	o	o
The HKCC contributes to a sense of community (perceived benefits, provider)	o	o	o	o	o	o	o
The HKCC enhances access to programs and	o	o	o	o	o	o	o

activities (perceived benefits, provider)							
The HKCC is effective in increasing knowledge of health behaviours (perceived benefits, provider)	o	o	o	o	o	o	o
The HKCC is effective in changing health behaviours (perceived benefits, provider)	o	o	o	o	o		o
The HKCC is effective in reducing childhood overweight and obesity in my community (perceived benefits, provider)	o	o	o	o	o	o	o

<u>Self-efficacy and self-proficiency</u>

13. **Please indicate the extent to which you agree or disagree with each of the following statements:**

	Strongly Agree	Agree	Neither agree nor disagree	Disagree	Strongly Disagree	Not applicable	Prefer not to answer
I feel confident in my ability to implement the HKCC in my community (self-efficacy, provider)	o	o	o	o	o	o	o
I have the necessary skills to engage with community partners (self-proficiency, provider)	o	o	o	o	o	o	o
I find it challenging to engage	o	o	o	o	o	o	o

community partners to participate in the HKCC (self-proficiency, provider)							
I believe we can achieve the goals of the HKCC in my community (self-proficiency, provider)	o	o	o	o	o	o	o

Please provide details about the strategies you used to engage with community partners:
____
____
____

Capacity Building and Support

<u>Training and technical assistance (For LPM)</u> (Training and technical assistance)

14. **Please indicate the extent to which you agree or disagree with each of the following statements:**

	Strongly Agree	Agree	Neither agree nor disagree	Disagree	Strongly Disagree	Not Applicable	Prefer not to answer
The Project Manager guidance document clearly defines my role and responsibilities	o	o	o	o	o	o	o
The MOHLTC webinar topics have been relevant to HKCC implementation	o	o	o	o	o	o	o
The Healthy Kids Resource Centres (HKRC) webinar topics have been relevant to HKCC implementation							
The resources/ materials provided by the HKRC were useful	o	o	o	o	o	o	o

HKRC support has addressed my specific questions or concerns	o	o	o	o	o	o	o
The Source (Online Networking Platform) was useful for sharing with and learning from other LPMs	o	o	o	o	o	o	o
Networking via email was useful to consult with and learn from other LPMs.							
The theme-based tool kits and fact sheets provided by the Ministry are useful	o	o	o	o	o	o	o
1:1 support from my contact at the Ministry has addressed my specific questions or concerns	o	o	o	o	o	o	o
I know where to find the necessary resources related to HKCC implementation	o	o	o	o	o	o	o
I have the information I need to complete Ministry reporting requirements	o	o	o	o	o	o	o
We can access other resources from non-HKCC sources	o	o	o	o	o	o	o

COMMUNITY CHARACTERISTICS

15. **Please indicate the extent to which you agree or disagree with each of the following statements, as they relate to your community:**

	Strongly Agree	Agree	Neither agree nor disagree	Disagree	Strongly Disagree	Don't Know	Not Applicable
There is strong local political commitment to support the HKCC (politics)	o	o	o	o	o	o	o
There is strong support from local community partners for HKCC implementation (based on community pilot comments)	o	o	o	o	o	o	o
There is sufficient funding from the province for HKCC implementation (funding)	o	o	o	o	o	o	o
There are sufficient policies in place in my community that support implementation of HKCC initiatives (policy)	o	o	o	o	o	o	o

16. **Did your community receive extra financial support for HKCC implementation in your community? (FOR LPM)** (funding)

    a.Yes (please describe): ____
    b.No
    c.Don't know
    d.Not applicable
    e.Prefer not to answer

17. **Did your organization contribute extra funding to support HKCC activities (e.g., planning, implementation, or evaluation activities)?** (funding)

    a.Yes (please describe): ____
    b.No
    c. Don't know
    d.Not Applicable
    e.Prefer not to answer

18. **Did your organization provide in-kind support for the HKCC in your community? (e.g., printing, space, supplies, etc.) (FOR LSC)**

    a.Yes (please describe): ____
    b.No
    c.Don't know
    d.Not applicable
    e.Prefer not to answer

<u>Community Structures or Partnerships (community support)</u>

Community-based health promotion interventions take place within settings, and include self-organizing activities of individuals. These activities often culminate in associations, unions, churches, and self-help groups. For the HKCC, having multiple partners in different sectors can help drive program success in your community.

19. **Please indicate the extent to which you agree or disagree with each of the following statements:**

	**Strongly Agree**	**Agree**	**Neither agree nor disagree**	**Disagree**	**Strongly Disagree**	**Don't Know**	**Prefer not to answer**
We have developed links with pre-existing community structures (community structures)	o	o	o	o	o	o	o
As a result of the HKCC, we have created new community structures and networks (community structures)	o	o	o	o	o	o	o
We are or have networked with diverse sectors to gain support for the HKCC (community structures)	o	o	o	o	o	o	o
We link with community groups or organizations to spread key messages from the HKCC in the	o	o	o	o	o	o	o

community (community structures)							
We link with community groups or organizations to expand their own programs to include HKCC initiatives (integration of new programming, innovation)	o	o	o	o	o	o	o

20. **All communities were encouraged to form and sustain multi-sectoral partnerships to support the planning and implementation of the HKCC. Please indicate the extent to which you agree or disagree with each of the following statements, as they relate to your community partnerships**:
    (IO: perceived benefits of innovation-community partnerships):

**As a result of the HKCC,**	**Strongly Agree**	**Agree**	**Neither agree nor disagree**	**Disagree**	**Strongly Disagree**	**Don't Know**	**Prefer not to answer**
Trust among partners has increased	o	o	o	o	o	o	o
Coordination among partners has improved	o	o	o	o	o	o	o
Collaboration on spin-off projects has increased	o	o	o	o	o	o	o
Partnerships formed from the HKCC are likely to continue after HKCC funding ends	o	o	o	o	o	o	o
Effective communication channels among partners have formed	o	o	o	o	o	o	o
There is a shared HKCC-related vision and shared goals among partners	o	o	o	o	o	o	o

21. My community partnered with the private sector on the HKCC:
    a. No, we do not have any private partners on the HKCC
    b. Yes, we have informal private partners on the HKCC (without a partnership charter)
    c. Yes, we have formal private partners on the HKCC (with a partnership charter)
    d. Prefer not to answer

    If you answered 'Yes' to having any private partners, please indicate the number of partners:

____

____

____

Sustainability of the HKCC Program

Program maintenance and sustainability has been defined as: “The capacity to continue to deliver a particular program through a network of agencies, in addition to, or instead of, the agency which initiated the program.” The next few questions are related to planning for sustainability.

	**To little or no extent**	**(sliding scale)**					**To a very great extent**
22. The HKCC program is integrated into the operations of the host organization (i.e., public health unit, City)	o	o	o	o	o	o	o
23. The HKCC Program is integrated into partner organizations							
24. The HKCC program in your community has a sustainability plan	o	o	o	o	o	o	o
25. The HKCC program has increased community awareness of child health behaviours							
26. Diverse community organizations are invested in the ongoing success of the program							
27. The HKCC has strong public support in my community							

28. Community champions exist who strongly support the program							
29. Community members (i.e., children, parents/caregivers) are engaged with the program							
30. Organizational systems are in place to support the various program needs beyond the HKCC funding period							

31. My community has conducted or plans to conduct local evaluation:
    a. Yes
    b. No
    c. Don't know
    d. Prefer not to answer

32. Does your community plan to continue programs established during the HKCC beyond the end of the funding period?
    a. Yes, all programming
    b. Yes, some programming
    c. No
    d. Unsure

33. What proportion of partnerships formed do you think will be maintained beyond the funding period?
    a. All
    b. Most
    c. About half
    d. Some
    e. None

34. Can you please explain why this is the case? ____

Implementation of Themes

35. Please rank ~~rate~~ the four themes based on ease with which they were implemented ~~(sliding scale from not difficult to very difficult, with NA as an option).~~
    a. Run. Jump. Play. Everyday.
    b. Water does Wonders

    c. Choose to Boost Veggies and Fruit
    d. Power off and Play

(with an open text field for explanation)

36. Please rate the four themes based on the ease with which partnerships were developed (sliding scale from not difficult to very difficult)
    a. Run. Jump. Play. Everyday.
    b. Water does Wonders
    c. Choose to Boost Veggies and Fruit
    d. Power off and Play

LPM ONLY

37. As the LPM, do you plan to continue being involved in programs developed during the HKCC beyond the funding period?

LSC Members Only

38. Will you continue working with other members of the LSC on initiatives developed during the HKCC after the end of the funding period?

Understanding Local Steering Committee (organization characteristics-delivery system)

39. **Please indicate the extent to which you agree or disagree with each of the following statements, as they relate to your community's Local Steering Committee:**

	**Strongly Agree**	**Agree**	**Neither agree nor disagree**	**Disagree**	**Strongly Disagree**	**Not Applicable**	**Prefer not to answer**
We developed sub/working groups to ensure relevance of skills and work							
There is good communication among members of the LSC (communication)	o	o	o	o	o	o	o

The LSC has a process for decision-making (shared decision-making)	o	o	o	o	o	o	o
	o	o	o	o	o	o	o
There is strong leadership on the LSC	o	o	o	o	o	o	o
Overall, I am satisfied with the way the LSC functions (positive organization climate)	o	o	o	o	o	o	o
Community champion(s) have supported the implementation of HKCC activities in our community	o	o	o	o	o	o	o

Implementation fidelity and adaptation (FOR LPM) (implementation outcomes)

Fidelity is defined as the extent to which the HKCC intervention is implemented as planned. Adaptation refers to changes to the HKCC program, often made to reflect the local context.

40. Thinking about the Theme 2 message ("Water Does Wonders"):
    a. My community used the same message to promote theme 2
    b. My community adapted the message to promote theme 2
    c. My community did not use the message to promote theme 2
    d. Prefer not to answer

41. If your community adapted the message (option 2), please let us know how and why you adapted the message (below):

____

____

____

42. Thinking about the Theme 3 message (“Choose to Boost Veggies and Fruit”):
    a. My community used the same message to promote theme 3
    b. My community adapted the message to promote theme 3
    c. My community did not use the message to promote theme 3
    d. Prefer not to answer

43. If your community adapted the message (option 2), please let us know how and why you adapted the message (below):

____

____

____

44. Thinking about the Theme 4 message (“Power Off and Play”):
    a. My community used the same message to promote theme 4
    b. My community adapted the message to promote theme 4
    c. My community did not use the message to promote theme 4
    d. Prefer not to answer

45. If your community adapted the message (option 2), please let us know how and why you adapted the message (below):

____

____

____

Social Marketing

46. Does your community use any of the following social media accounts?
    a. Facebook
    b. Twitter
    c. Instagram
    d. Pinterest
    e. Other (please describe)
    f. No

47. Does your community piggy-back to another social media account? (for example, does your municipality have an account through which you do HKCC or theme-related promotion)? If so, please provide details below:

    ____

    ____

    ____

    ____

48. How did you use the social marketing materials provided by MOHLTC in your delivery of the HKCC?
    a. My community used the materials provided
    b. My community adapted some of the materials as appropriate

c. My community adapted most of the materials
d. We did not use the materials provided
e. Prefer not to answer

49. In addition to using the HKCC brand, my community gave HKCC a different brand or name to promote the HKCC program:
    a. Yes
    b. No
    c. Don't know
    d. Prefer not to answer

50. If you chose yes, please let us know the brand your community used and your reason for doing so.

____

____

____

<u>Community Outcomes of HKCC</u> (implementation outcomes – added based on community's pilot feedback)

51. **Please indicate the extent to which you agree or disagree with each of the following statements:**

**As a result of HKCC,**	**Strongly Agree**	**Agree**	**Neither agree nor disagree**	**Disagree**	**Strongly Disagree**	**Not Applicable**	**Prefer not to answer**
There are more programs/activities that promote healthy behaviours	o	o	o	o	o	o	o
Supportive environments have been built to promote healthy behaviours	o	o	o	o	o	o	o
New local policies have been developed to support healthy behaviours	o	o	o	o	o	o	o
Community partnerships have been formed or strengthened	o	o	o	o	o	o	o
Knowledge of health behaviours has increased in my community	o	o	o	o	o	o	o

52. Other comments:

____
____
____

OPEN-ENDED QUESTIONS

53. **Please describe any positive experiences related to the implementation of HKCC initiatives in your community.**

____
____
____

54. **Please describe any barriers related to the implementation of HKCC initiatives in your community.**

____
____
____

55. **How could the HKCC program be improved to better serve your community needs? Please provide your suggestions below.**

____
____
____

Tell us about yourself

56. **Please specify your gender:**
    a. Female
    b. Male
    c. Trans
    d. Other (Please specify)
    e. Prefer not to answer

57. To which age group do you belong:

a. Less than 18 years
b. 18-29 years
c. 30-39 years
d. 40-49 years
e. 50-59 years
f. 60-64 years

g. 65 years or over
h. prefer not to answer

58. How many years of experience do you have working within your community? (FOR LPM)

a. 1 year or less
b. 2-5 years
c. 6 to 10 years
d. 11 to 20 years
e. more than 20 years
f. prefer not to answer

59. How many years of experience do you have in relationship management or working with multi-sectoral partners? (FOR LPM)

a. 1 year or less
b. 2 to 5 years
c. 6 to 10 years
d. 11 to 20 years
e. more than 20 years
f. prefer not to answer

60. How many years of experience do you have in managing large projects with programming/policy elements? (FOR LPM)

a. 1 year or less
b. 2 to 5 years
c. 6 to 10 years
d. 11 to 20 years
e. more than 20 years
f. prefer not to answer

61. How many years of experience do you have in program evaluation or data collection? (FOR LPM)

a. 1 year or less
b. 2 to 5 years
c. 6 to 10 years
d. 11 to 20 years
e. more than 20 years
f. prefer not to answer

62. How many years of experience do you have in social marketing? (FOR LPM)

1. 1 year or less
2. 2 to 5 years
3. 6 to 10 years
4. 11 to 20 years
5. more than 20 years

6. prefer not to answer
