# Supplementary material for: An early implementation assessment of Ontario’s Healthy Kids Community Challenge: results from a survey of key stakeholders
Source: BMC Public Health. 2019 Nov 27;19:1568. doi: 10.1186/s12889-019-7704-2 (PMC6880511; doi:10.1186/s12889-019-7704-2)
Supplement: Supplementary file 3 — Additional file 3. Summary of qualitative results, including themes, number of mentions and selected quotations. [file 12889_2019_7704_MOESM3_ESM.docx]

**Additional File 3:** Summary of qualitative results, including themes, number of mentions and selected quotations (Durlak and DuPre, 2008).

| **Implementation Framework Categories** | **Themes & Sub-Themes** | **Number of Mentions (Count)** | **Selected Quotations** |
| --- | --- | --- | --- |
| **a) Community Level Factors** | - **Funding** (e.g., in-kind, funding for programming, activities, sustainability, evaluation) - **EPODE or provincial HKCC** (e.g., timelines, evidence) - **Physical environment** (e.g., geography, transportation, infrastructure) - **Policy** (e.g., municipal, provincial) - **Politics** (e.g., political support for HKCC, political structure) | 79  43  32  7  6 | “*Free use of municipal grounds, storage space and community centre, free printing and supplies, volunteer hours for equipment assembly and installation, staff time for equipment purchasing and lending hub set up.*” [**Funding**]  “*Time, not only to organize the action plan, but to have our partners organize and implement the actual projects in the action plan within the 9 months. Some of our projects are quite large in scale and require A LOT of preparation. As a result, 9 months is not enough time to start and finish some of our projects…*” [**EPODE or provincial HKCC**]  “*There are agencies and organizations we can work with but they do not cover the whole city so there are some groups of vulnerable populations which are harder to reach.*” [**Physical environment**] |
| **b) Provider Characteristics** | - **Perceived benefits of HKCC** (e.g., community outcomes, knowledge or awareness of HKCC health behaviours, health behaviour changes, changes in healthy weights) - **LPM or LSC member skills and expertise** - **Perceived need for HKCC** | 39  3  2 | “*It has also been great to see the amount of media attention that HKCC has attracted and the 'buzz' it is generating. Kids and their families are recognizing our HKCC branding and they are excited about our activities.*” [**Perceived benefits of HKCC**]  “*Our committee has good representation/participation from those with local expertise in community development, program planning and evaluation.*” [**LPM or LSC member skills and expertise**] |
| **c) Characteristics of the HKCC (innovation)** | - **Adaptability of the HKCC** (e.g., population reach, program adaptations) - **Compatibility** (e.g., community acceptance of HKCC, host organization support) - **Evidence to inform local HKCC** | 61  13  2 | “*Within my community we have implemented a program that directly targets at-risk and marginalized youth by offering them leadership training and a substantive voice at the planning table.*” [**Adaptability of the HKCC**]  “*Currently there are not any standards that communities must uphold for children's health. The goal is for the health of children to become a priority and for communities to commit to providing the necessary supports and positive health environments for the children in our region.*” [**Compatibility**] |
| **d) Prevention Delivery System** | - **Group relationships between stakeholders** (e.g., group relationships, collaboration, commitment, participation) - **Specific practices and processes** (e.g., decision-making, communication, governance model, working groups, meeting schedules - **Specific role considerations** (e.g., LPM leadership, workload of LPM, political champion, community champion, Chair or Co-Chair) | 97  17  7 | “*We have seen community organizations with similar programs/initiatives willing to come together to discuss how they can, with some compromises, work together to strengthen their programs/services objectives. We have seen some creative projects arise and become stronger through collaborative evolution, via the idea generating forums we host around each HKCC theme as it is revealed*.” [**Group relationships between stakeholders: collaboration**]  “*Despite my organization having significant experience with peer mentoring and training and despite offering those services multiple times, we were never asked to help in this regard. Decisions were made away from the planning table about resource allocation and no updates on the peer mentoring initiative were ever made available to the community planners.*” [**Specific practices and processes**] |
